# A survey on cellular RNA editing activity in response to *Candida albicans* infections

**DOI:** 10.1186/s12864-017-4374-2

**Published:** 2018-01-19

**Authors:** Yaowei Huang, Yingying Cao, Jiarui Li, Yuanhua Liu, Wu Zhong, Xuan Li, Chen Chen, Pei Hao

**Affiliations:** 1 Key Laboratory of Molecular Virology and Immunology, Institute Pasteur of Shanghai, Chinese Academy of Sciences, Shanghai, 20031 China; 2 Institute of Infectious Diseases, Beijing Ditan Hospital, Capital Medical University, Beijing, 100102 China; 3 Beijing Key Laboratory of Emerging Infectious Diseases, Beijing, 100102 China; 4 National Engineering Research Center For the Emergence Drugs, Beijing Institute of Pharmacology and Toxicology, Beijing, 100850 China; 5 Key Laboratory of Synthetic Biology, CAS Center for Excellence in Molecular Plant Sciences, Institute of Plant Physiology and Ecology, Shanghai Institutes for Biological Sciences, Chinese Academy of Sciences, Shanghai, 20032 China

**Keywords:** ADAR, A-to-I RNA-editing, *Candida albicans*, Infection, Fungi-host interaction

## Abstract

**Background:**

Adenosine-to-Inosine (A-to-I) RNA editing is catalyzed by the adenosine deaminase acting on RNA (ADAR) family of enzymes, which induces alterations in mRNA sequence. It has been shown that A-to-I RNA editing events are of significance in the cell’s innate immunity and cellular response to viral infections. However, whether RNA editing plays a role in cellular response to microorganism/fungi infection has not been determined. *Candida albicans*, one of the most prevalent human pathogenic fungi, usually act as a commensal on skin and superficial mucosal, but has been found to cause candidiasis in immunosuppression patients. Previously, we have revealed the up-regulation of A-to-I RNA editing activity in response to different types of influenza virus infections. The current work is designed to study the effect of microorganism/fungi infection on the activity of A-to-I RNA editing in infected hosts.

**Results:**

We first detected and characterized the A-to-I RNA editing events in oral epithelial cells (OKF6) and primary human umbilical vein endothelial cells (HUVEC), under normal growth condition or with *C. albicans* infection*.* Eighty nine thousand six hundred forty eight and 60,872 A-to-I editing sites were detected in normal OKF6 and HUVEC cells, respectively. They were validated against the RNA editing databases, DARNED, RADAR, and REDIportal with 50, 80, and 80% success rates, respectively. While over 95% editing sites were detected in Alu regions, among the rest of the editing sites in non repetitive regions, the majority was located in introns and UTRs. The distributions of A-to-I editing activity and editing depth were analyzed during the course of *C. albicans* infection. While the normalized editing levels of common editing sites exhibited a significant increase, especially in Alu regions, no significant change in the expression of ADAR1 or ADAR2 was observed. Second, we performed further analysis on data from in vivo mouse study with *C. albicans* infection. One thousand one hundred thirty three and 955 A-to-I editing sites were identified in mouse tongue and kidney tissues, respectively. The number of A-to-I editing events was much smaller than in human epithelial or endothelial cells, due to the lack of Alu elements in mouse genome. Furthermore, during the course of *C. albicans* infection we observed stable level of A-to-I editing activity in 131 and 190 common editing sites in the mouse tongue and kidney tissues, and found no significant change in ADAR1 or ADAR2 expression (with the exception of ADAR2 displaying a significant increase at 12 h after infection in mouse kidney tissue before returning to normal).

**Conclusions:**

This work represents the first comprehensive analysis of A-to-I RNA editome in human epithelial and endothelial cells. *C. albicans* infection of human epithelial and endothelial cells led to the up-regulation of A-to-I editing activities, through a mechanism different from that of viral infections in human hosts. However, the in vivo mouse model with *C. albicans* infection did not show significant changes in A-to-I editing activities in tongue and kidney tissues. The different results in the mouse model were likely due to the presence of more complex in vivo environments, e.g. circulation and mixed cell types.

**Electronic supplementary material:**

The online version of this article (10.1186/s12864-017-4374-2) contains supplementary material, which is available to authorized users.

## Background

A-to-I RNA editing is a hydrolytic deamination reaction at the C6 position of adenine base occurring on double stranded RNAs, which is catalyzed by the adenosine deaminases acting on RNA (ADAR) family of enzymes [1, 2]. Inosines (I) that are converted from adenosines (A), will be translated as guanosine by ribosome, which could result in altered sequence in protein products. ADAR1, ADAR2, and ADAR3 are three members of the ADAR gene family found in human [3]. They consisted of two to three double strand RNA-binding domains (dsRBD) and a deaminase domain in the C-terminal region. ADAR1 has two isoforms, a constitutively expressed ADAR1 p110 and an IFN-induced ADAR1 p150 [4]. As shown in previous studies, dysregulated RNA editing is associated with numerous human diseases, including cancers like acute myeloid leukemia (AML), Astrocytoma, or hepatocellular carcinoma [5–7], and neurological or psychiatric disorders like Amyotrophic lateral sclerosis (ALS), epilepsy, depression, and suicide [8]. Mutant ADARs were also related to Aicardi-Goutières syndrome (AGS) [9, 10] and dyschromatosis symmetrica hereditaria (DSH) [11, 12].

Roles of A-to-I RNA editing were shown to be involved in innate immune response in virus-infected hosts, which displayed either antiviral or proviral activities by inducing changes that affect how viruses interact with their hosts [13, 14]. The RNA editing machinery may contribute to the hyper-mutation and diversification of RNA virus like noroviruses (NoVs) [15]. ADAR-mediated editing of the viral genome during replication was found to be partially responsible for the high mutation rates of NoVs, in addition to the low replication fidelity of RNA virus polymerases. Ebolavirus (EBOV) and Marburgvirus (MARV) were also found to contain novel filovirus-host interactions in infected hosts by addition of non-template-encoded residues within the EBOV glycoprotein (GP) or MARV nucleoprotein (NP), which was induced by apparent RNA-editing activities [16]. Although a lot of studies have been focused on the function of RNA editing both in hosts and microorganisms [17–19], no systematic study has been found on the function of RNA editing in mammalian hosts during the infection of fungi. Since innate immunity is known to response to bacteria/fungi infections, it is important to investigate whether the RNA editing mechanism is involved in cellular response to microorganism infection.

*Candida albicans* is one of the most prevalent human pathogenic fungi. It grows as yeast or filamentous cells and can cause the infection candidiasis in humans, which was reported as the 4th leading cause [20] of bloodstream infections acquired during hospital stay and incurred a very high mortality and a severe morbidity in the U.S. [21, 22]. Many nutrient factors, iron and zinc sequestration, pH, temperature, and osmotic pressure are crucial for the growth of commensal *C. albicans* in human hosts [22]. Numerous studies have revealed the complex interplay between fungal virulence factors utilized by *C. albicans* and the host immune response [23–27]. The state of *C. albicans* growing and coexisting with a human host without causing any symptoms of disease, is very much dependent on the immunological cross-talk between *C. albicans* and the human innate immune system. Alterations in the host can replace commensal factors with virulence attributes once the pathogenicity is induced. As a result, fungal cells adhering to the host epithelial cells begins invasion by host endocytosis, then active penetration and directs damage follows. Whether RNA editing plays a role in *C. albicans*-host immune system cross talk as a part of the innate immune response system remains unknown. In the current study, to investigate the A-to-I RNA editing events in *C. albicans* infections*,* a big-data analysis was performed using community RNA-seq data sets generated from human endothelial cells and oral epithelial cells during in vitro infection with 2 *C. albicans* stains, WO1 or SC5314 and from tongue and kidney tissues from mouse model of hematogenously disseminated candidiasis (HDC) [28]. This work represents the first comprehensive analysis of A-to-I RNA editome in human epithelial and endothelial cells, and a possible role of A-to-I RNA editing in *C. albicans* infection was implicated with in vitro infection data.

## Results

### Global profiles of A-to-I RNA editing events in human epithelial and endothelial cells

RNA-seq data were obtained from oral epithelial cells (OKF6 cell line) and primary human umbilical vein endothelial cells (HUVEC cell line) infected with SC5314 or WO1 strains and collected at 1.5, 5, and 8 h post infection [28]. The control uninfected OKF6 and HUVEC cell lines were used as control. (For the details of the sample information, see Additional file 1). To explore the influence of *C. albicans* infection on RNA editing of human epithelial and endothelial cells, we first analyzed the RNA-editing activities in human epithelial cells (OKF6 cell line) and endothelial cells (HUVEC cell line), which have no reported studies to date. We had 487 and 300 million paired-end RNA-seq reads (2X 100 bp) for OKF6 and HUVEC, respectively, for which the mapped reads were 409 and 268 million (see Additional file 2).

**Fig. 1:**
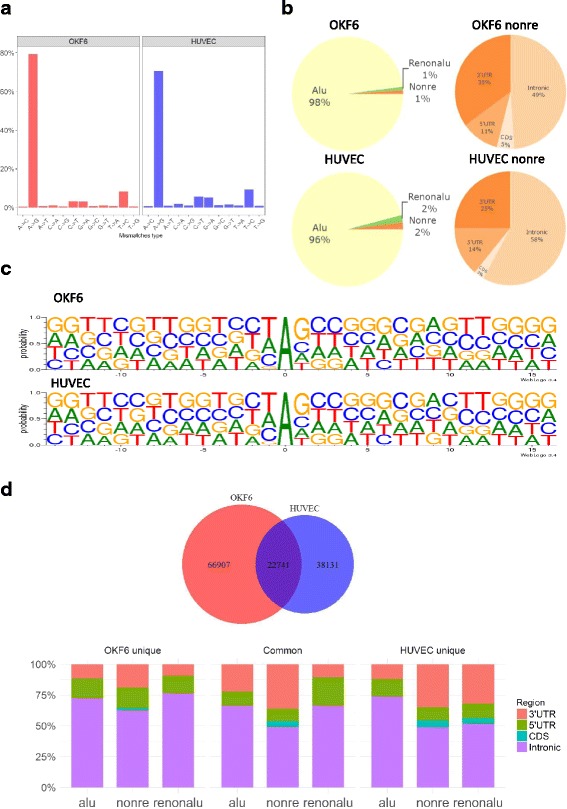
Characterization of A-to-I RNA-editing events in human epithelial and endothelial cells. **a** Overview of 12 types of mismatches identified in epithelial and endothelial cells. **b** Distribution of epithelial and endothelial RNA editing sites in Alu, non repetitive (Nonre), repetitive non Alu (Renonalu) region (left panel) and genomic distribution of RNA editing sites in none repetitive region (right panel). 3’UTR, three prime untranslated region. 5’UTR, five prime untranslated region. CDS, coding DNA sequence. **c** The pattern of the 15 flanking bases around editing sites in epithelial and endothelial cell. **d** Comparison between the RNA editing sites of epithelial and endothelial cells

We applied a pipeline modified from previous studies [29–31] to identify RNA-editing sites. Collectively, we obtained 89,648 and 60,872 nucleotide discrepancy sites for OKF6 and HUVEC cells, respectively (see Additional file 3). The A-to-I (shown as A-to-G) RNA editing sits contributed to most of the DNA-RNA alteration (Fig. 1a). The identified A-to-I RNA-editing sites were checked against the annotated RNA editing databases DARNED [32], RADAR [33] and REDIportal [34], and were validated with 50, 80, and 80% success rates, respectively (see Additional file 4).

As shown in Fig. 1b, more than 95% A-to-I editing sites were detected in Alu region in accordance with previously studies [30, 35], and the majority of the editing sites in non repetitive region are located in intronic and untranslated regions (UTR). The pattern of the 15 flanking bases of editing sites were similar to previous results [35–38]. The nucleotides had a strong preference on upstream and downstream positions of the editing sites with G depletion in −1 position and G enrichment in +1 position (Fig. 1c).

Comparison of the editing sites identified in two cell types (Fig. 1d), 22,741 A-to-G editing sites commonly occurred in epithelial and endothelial cells, whereas 66,907 and 38,131 editing sites were uniquely identified in OKF6 and HUVEC cells, respectively. Their distributions were further defined for different genomic regions (Fig. 1d), with the majority of editing sites located in intronic and untranslated region. Unique editing sites of non Alu region in OKF6 cells were found more frequently in intronic than those in HUVEC cells. In contrast, there are more editing sites detected in 3’ UTR of HUVEC cell than in OKF6 cell. The same happened in repetitive non Alu region as well. Taken together, the RNA editome of epithelial and endothelial cells displayed some similar characteristics, but majority of editing events were unique to each. This represents the first comprehensive analysis of human epithelial and endothelial cell RNA editome.

### Changes in RNA editing in human epithelial and endothelial cells infected with *C. albicans*

**Fig. 2:**
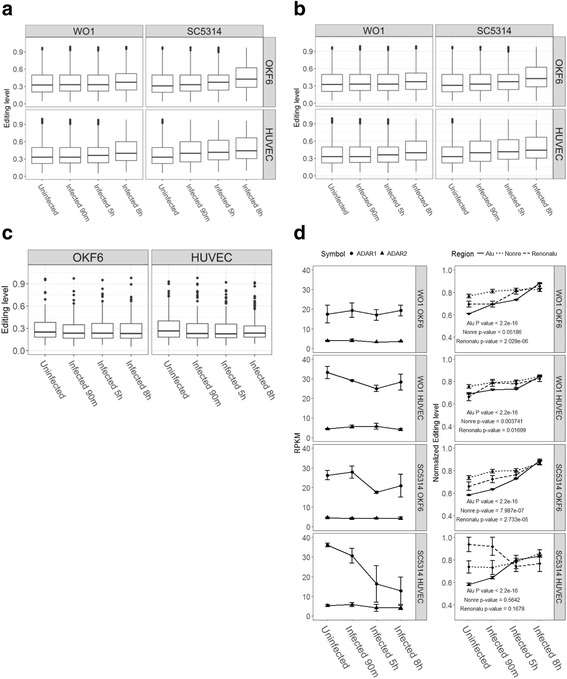
Pattern of RNA editing during the course of infection in epithelial and endothelial cells. **a** Distribution of editing level in different infection conditions. **b** Distribution of editing level in stable expression genes. **c** Distribution of editing level in significant differential expression genes. **d** Pattern of ADAR genes expression and the normalized editing level of common editing site

To characterize the effects of *C. albicans* infection on A-to-I RNA editing in human epithelial and endothelial cells, we investigated the changes in editing activities during the course of infection. When mean editing level was used to measure the changes of editing activity (Fig. 2a, and see Additional files 5 and 6), it ranged from 0.36 to 0.44 in epithelial cells, and from 0.38 to 0.47 in endothelial cells. Upward changes were observed at later time points in both during the course of infection. We then looked more carefully at the pattern of A-to-I editing sites located in either stably expressed genes or differentially expressed ones. For stably expressed genes (Fig. 2b, and see Additional file 7), the mean editing level ranged from 0.28 to 0.31 in epithelial cells, and from 0.27 to 0.34 in endothelial cells. For differentially expressed genes (Fig. 2c, and see Additional file 8), the mean editing level spanned from 0.23 to 0.25 in epithelial cells compared to from 0.22 to 0.27 in endothelial cells. So for the subsets of stably and differentially expressed genes, no significant changes in editing pattern can be detected in epithelial and endothelial cells with WO1 or SC5314 infections.

As shown previously in human cancers [5, 39, 40], the normalized editing levels were found to be correlated to ADAR gene expression. Thus, we investigated the correlation between ADARs expression (Fig. 2d left panels) and the normalized editing levels of common editing sites (Fig. 2d right panels). Expression of ADAR1 and ADAR2 genes varied, whereas ADAR3 gene expression was not detectable in human epithelial and endothelial cells. While the expression of ADAR1 showed a decline in SC5314 infected HUVEC cell lines, no significant changes in either ADAR1 or ADAR2 expression were detected during the infection. In comparison, the normalized editing levels of the common editing sites (Fig. 2d right panel) exhibited an significantly upward changing patterns in all infection conditions, especially in Alu regions. The increased activities in A-to-I RNA editing over the course of infection can not be explained by the expression level of ADAR1 or ADAR2 (see Additional file 9). This observation differs from those of previous studies [5, 39, 40] and our new research data submitted with this manuscript in parallel, which suggests that the A-to-I editing activities were up-regulated by a different mechanism in *C. albicans* infection in human epithelial and endothelial cells.

### Profiles of A-to-I RNA editing events in mouse model in vivo

**Fig. 3:**
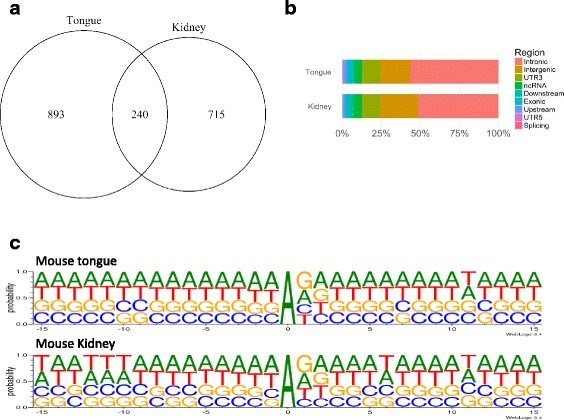
Charactrization of RNA-editing sites of mouse tissues. **a** Overlap of RNA-editing sites between mouse tongue and kidney. **b** Distribution of RNA-editing sites in mouse genomic regions. **c** Pattern of the 15 flanking bases of RNA-editing sites in mouse tongue and kidney tissues

We used *C. albicans* infected mouse model to further analyze RNA editing changes in epithelial and endothelial cells during infection. The RNA-seq data from mouse tongue and kidney tissues were available from a recent study [28]. 584 and 605 million mapped reads were mapped to the mouse genome, respectively (see Additional file 10). We identified 1133 and 955 discrepancy editing sites in the mouse tongue and kidney RNA-seq data, respectively (Fig. 3a, and see Additional file 11), for which A-to-G mismatches were the majority. Compared to human epithelial and endothelial A-to-I editing sites, the mouse editing sites had a lower overlap between tongue and kidney tissues (Fig. 3a). We then annotate these editing sites (Fig. 3b), and found introns, intergenic regions of genes, and 3’-UTR were the most enriched regions for A-to-I editing sites for these tissues. Next, we analyzed the upstream and downstream 15-bases sequence flanking editing sites (exhibited by WebLogo3) (Fig. 3c). The flanking sequences of RNA editing sites in mouse tissues were similar to those of human with a depletion of G in 5′ and an increase in 3′ of editing sites, but the G depletion was less in mouse than in human. These data represents the first A-to-I RNA editome from mouse tongue and kidney tissues.

### Changes of A-to-I RNA editing in mouse model infected with *C. albicans* in vivo

**Fig. 4:**
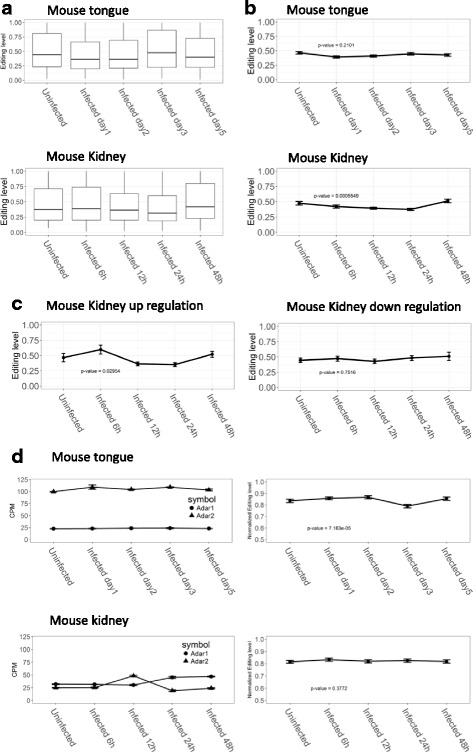
Pattern of RNA editing sites in mouse tissues. **a** Distribution of editing level in mouse tongue and kidney tissues. **b** Pattern of editing level in stable expression genes. **c** Pattern of editing level in differentially expression genes. **d** Pattern of ADAR genes expression and normalized editing level of common editing sites

To illustrate the changes of RNA editing in mouse model infected with *C. albicans* in vivo, we analyzed the distribution of editing level across different mouse tissues. The mean editing level of sites in tongue tissues were between 0.40 to 0.52, and that of editing sites in kidney tissues were between 0.41 to 0.50 (Fig. 4a, and see Additional files 12 and 13). We then looked at the pattern of editing levels in stably expressed and differentially expressed genes (Fig 4b). The editing sites located in stably expressed genes showed no significant changes in tongue tissues. For those sites in stably expressed genes in kidney tissue, the editing level decreased initially throughout the course of infection and returned to pre-infection level at 48 h post infection. For the editing sites located in differentially expressed genes, 212 and 194 sites were found in up- and down-regulated genes of kidney tissues, respectively (Fig. 4d), whereas only 17 sites are found in tongue tissues. In kidney tissues the editing level of sites in up-regulated genes increased significantly at 6 h post infection and then decreased at 12- and 24- h post infection before returning to normal at 48 h after infection. For editing sites in down-regulated genes in kidney tissues, the editing level remained un-changed along the different time points after infection.

Accordingly, we examined the relationship between ADAR gene expression and the editing level of A-to-I RNA editing sites (Fig. 4d, and see Additional file 14). We obtained 131 and 190 common editing sites in mouse tongue and kidney tissue, respectively. The ADAR genes showed no significant change in expression among tongue tissues. The ADAR2 gene, which was found to be highly expressed in brain tissues reported previously [8, 41], exhibited a high expression level in tongue. The ADAR2 gene had a significant increase at 12 h after infection and then decreased to pre-infection level at 24 h, whereas ADAR1 remained constant throughout. On the other hand, the normalized editing levels remained stable in kidney tissues along the course of infection, whereas in tongue tissues the normalized editing level had a slight drop on day three post infection and backed to normal on day 5. So neither the expression level of ADARs, nor the normalized RNA editing levels displayed significant changes during the course of *C. albicans* infection in mouse tongue and kidney tissues, except a short pulse with ADAR2 at 12 h post infection.

## Discussion

Being the most prevalent human pathogenic fungi, *C. albicans* accounts for 50–90% of all cases of candidiasis in humans. Host infection by *C. albicans* often initiates by transforming the unicellular yeast-like form of *C. albicans* into the multicellular filamentous form, thus becoming invasive to human epithelial and endothelial tissues. The complex interaction between *C. albicans* and the host immune system, including innate immunity, is a crucial, but little understood process. As RNA editing was previously shown to play significant roles in the innate immune response of hosts toward viral infections, it is important to understand whether RNA editing in human epithelial and endothelial cells plays a role in the innate immune response to *C. albicans* infection. To answer this question, the current study took a approach by integrating and re-analyzing two sets of data from previous experiments with human endothelial cells and oral epithelial cells during in vitro infection with 2 *C. albicans* stains, WO1 or SC5314, and with mouse model of hematogenously disseminated candidiasis (HDC) [28].

From our analysis, 89,648 and 60,872 A-to-I RNA editing events were identified for OKF6 and HUVEC cells, respectively. This represents the first comprehensive analysis of RNA editing editome in human epithelial and endothelial cells. The validity of these events was confirmed by cross-checking against the annotated RNA editing databases, DARNED, RADAR, and REDIportal. The significant fraction of newly identified A-to-I RNA editing events are interesting, as they represent editing sites specific to human epithelial and endothelial cells. Previously, functions of A-to-I RNA editing mediated by ADARs were discovered and documented mostly in neuronal receptors [42], and ion transporters [43]. Our results provide a useful reference for studying A-to-I RNA editing events in epithelial and endothelial cells, which points to likely significant functions in corresponding tissues.

To investigate the changes in A-to-I RNA editing events during the course of *C. albicans* infection, we measured their activities using the mean editing level of all events and the normalized editing levels of common editing sites across all time points. While the former displayed a slight upward trend of RNA editing activity during the course of infection, the latter showed a significant increase of RNA editing levels for sites located in the three different regions, Alu, Nonre, and Renonalu (Fig. 2d). Unexpectedly, the increased RNA editing levels were not associated with an increased expression of ADAR1 and ADAR2 genes (ADAR3 gene expression was not detectable) in human epithelial and endothelial cells. Previous studies [5, 39, 40] showed that increased A-to-I RNA editing activities were often associated with the up-regulated expression of ADARs. In the cases of (influenza) viral infected host cells, increased RNA editing activities were found to be accompanied by upregulation of ADARs through the innate immune pathway, newly revealed by our parallelly submitted paper. The observed difference between infection of influenza virus and that of *C. albicans* raises an interesting question about the mechanism of upregulated A-to-I editing activities in the case of *C. albicans* infection in human epithelial and endothelial cells, presenting a significant challenge for researchers in the field.

To further examine the effect of *C. albicans* infection on A-to-I RNA editing activities in epithelial and endothelial cells in vivo, we investigated a *C. albicans* infected mouse model to analyze RNA editing changes during infection. We identified a total of 1133 and 955 RNA-editing events in mouse tongue and kidney tissues, respectively. Due to the lack of Alu elements, mouse has a much lower number of A-to-I editing sites detected compared to human. Comparing to the sequence feature flanking editing sites in human cell lines, the 3′ G depletion in A-to-I RNA sites was much less in mouse tissues. In addition, these editing sites exhibited a significant tissue specificity. These data represents the first documented A-to-I RNA editome for mouse tongue and kidney tissues.

To illustrate the changes of RNA editing in mouse model infected with *C. albicans* in vivo, similarly we measured the RNA editing activities by the mean editing level of all events and by the normalized editing levels of common editing sites cross all time points for mouse tongue and kidney tissues. However, different from the results of *C. albicans* infected human epithelial and endothelial cells, the normalized editing levels remained stable in mouse kidney tissues along the course of infection, whereas the normalized editing level in tongue tissues dropped slightly on day three post infection before returning to normal. The different results from the in vivo mouse model were likely due to the more complex in vivo environments. Interactions between *C. albicans* and host animals are undoubtedly more complex because of the various factors exiting in in vivo environments, like the presence of circulating immune system cells. It is likely the immune cells modulate the response of epithelial and endothelial cells in the in vivo mouse model. It is also possible that the mixed cell types from mouse tongue and kidney tissues inundated the signals generated from epithelial and endothelial cells infected with *C. albicans*. Nonetheless, our current study suggested a likely roles of ADAR mediated A-to-I RNA editing in microorganism infections, like *C. albicans.* Future study may be pursued with additional microorganisms in pathogenic settings to understand the roles of RNA editing in innate immune response to whole scope of infectious agents.

## Conclusion

This work represents the first comprehensive analysis of A-to-I RNA editome in human epithelial and endothelial cells. It was proposed that *C. albicans* infection of human epithelial and endothelial cells up-regulated A-to-I editing activities through a mechanism different from that of viral infections of human hosts, such as influenza viral infections in our parallel study. However, a mouse in vivo study with *C. albicans* infection did not reveal significant changes in A-to-I RNA editing activities in tongue and kidney tissues. It is likely that in vivo infection with *C. albicans* experienced a different kinetics due to the presence of more complex environments, like circulation and immune cells.

## Methods

### Collection of RNA-Seq data

RNA-seq data for *C. albicans* infected cells or mouse tissues were collected from the NCBI Gene Expression Omnibus (GEO) under accession number GSE56093 and GSE67688 [28, 44]. Cell and animal treatment and infection with *C. albicans* were as described [28, 44]. Preparation of RNA-seq libraries and generation of RNA-seq data were performed according to standard Illumina protocols. The RNA-seq data and sample information were summarized in Supplementary Table S1.

### Processing and mapping of RNA-seq data

For RNA-seq data processing and mapping, our pipeline was modified from previous work [29–31, 45], from which small adjustment was made in order for it to work with our collected RNA-seq datasets. In brief, the Burrows-Wheeler algorithm (BWA) [46] was used for RNA-seq reads mapping on reference genomes (the human hg19 reference genome and mouse mm9 reference genome, bwa aln -t 4, bwa samse –n4). The PCR duplicates reads were removed by MarkDuplicates tools from Picard (version: picard-1.127; https://broadinstitute.github.io/picard/). Unmapped reads and those with mapping quality score lower than 20 were removed by Samtools (version 0.1.19, samtoosl view –bS –F 4 –q 20) [47].

### Calling and characterizing A-to-I RNA editing sites

The protocol we used to call A-to-I RNA editing sites was derived from that described previously. In brief, the RNA-seq data mapped to reference genomes were subject to variant calling by the GATK analysis tool [48]. The called variant sites were filtered by rigorous parameters as described [29, 30]. Briefly, we required variants identified both in human and mouse to be supported by at least three mismatched reads on editing sites to reduce false positives. Both A-to-G and T-to-C mismatches were combined and counted as A-to-I editing sites. To remove possibly false positive RNA editing events due to SNPs, human SNP (Build 141 by NCBI) and mouse SNP (Build 128 by NCBI) were downloaded using the UCSC table browser data retrieval tool [49] and were used to filter human and mouse RNA editing data, respectively.

### Computation of editing level and normalized editing level

Editing level is defined for an editing site as the number of reads with the edited base divided by the number of total reads mapped on the site. Normalized editing level is defined as the ratios of the editing levels at different time points to the maximum editing level for a specific editing site [50].

### Annotation of A-to-I RNA editing sites

We used CAVA [51] (that provides additional clinical information, like disease association, for base variants in human genes) to annotate the A-to-I RNA editing sites from human cell lines, and ANNOVAR [52] to annotate the RNA editing sites from mouse tongue and kidney tissues(CAVA is not developed to work for mouse genes). Sequence pattern around A-to-I RNA editing sites in human and mouse was delineated in two steps: 1) extracting the profile of up- and down-stream sequences (15 bases on each side) flanking editing sites using bedtools getfasta [53]; 2) visualizing the sequence context around RNA editing sites using WebLogo 3(weblogo –A dna –c classic –units probability –first-index −10) [54].

### Gene differential expression analysis

Level of expressed genes in RPKM (Reads Per Kilobase per Million mapped reads) was estimated from RNA-seq mapping results as described [28]. Briefly, HISAT2 [55] was used to map reads on reference genomes, HTSeq [56] was used to count mapped reads for expressed genes, and edgeR [57] was used to perform gene differential expression analysis. Differentially expressed genes were defined as fold-change greater than 2 and false discovery rate (FDR) smaller 0.05. All RNA-seq reads were first trimmed by Trimmomatic-0.32 [58] with parameters: HEADCROP = 10, SLIDINGWINDOW = 4:20 and MINLEN = 36). In addition, duplicated reads were removed by Picard.

### Statistics

R package Venn Diagram [59] was used to calculate and draw the overlapping between our identified RNA editing sites and those included in the databases: DARNED, RADAR and REDIportal. R package ggplot2 [60] was used for plotting other figures. For statistics testing with distribution of A-to-I RNA editing data, the nonparametric test, Kruskal-Wallis rank sum test, was performed. For correlation analysis between ADAR gene expression and normalized RNA editing levels, the Spearman’s rank correlation coefficient was computed with R.

## Additional files

Additional file 1: The data sets information of collected RNA seq sample. (XLS 36 kb)

Additional file 2: RNA editing sites of OKF6 and HUVEC cell lines. (XLS 29 kb)

Additional file 3: Mouse organ or tissues RNA-seq mapping results. (XLS 13269 kb)

Additional file 4: Cell line RNA-editing Sites overlap with database. (XLS 27 kb)

Additional file 5: The number of RNA editing sites in OKF6 and HUVEC infection with different time point and strains. (XLSX 9 kb)

Additional file 6: Editing level of RNA editing sites in OKF6 and HUVEC infection with different time point and strains. (XLS 28 kb)

Additional file 7: Editing level of RNA editing sites locate in stable expression genes. (XLSX 9 kb)

Additional file 8: Editing level of RNA editing sites locate in differentially expression genes. (XLSX 8 kb)

Additional file 9: Correlation test between the genes expression of ADAR and the normalized editing level of common editing sites. (XLS 30 kb)

Additional file 10: Mouse organ or tissues RNA-seq mapping results. (XLS 36 kb)

Additional file 11: RNA editing sites of mouse organ or tissues. (XLS 215 kb)

Additional file 12: The number of RNA editing sites with mapped bases in mouse model infection with *C. albicans*. (XLS 26 kb)

Additional file 13: Distribution of RNA editing level of mouse organ or tissue. (XLS 27 kb)

Additional file 14: Correlation test between the genes expression of Adar and the normalized editing level of common editing sites in mouse. (XLS 28 kb)

## References

[1] Bass BL (2002). RNA editing by adenosine deaminases that act on RNA. Annu Rev Biochem.

[2] Barraud P, Allain FH (2012). ADAR proteins: double-stranded RNA and Z-DNA binding domains. Curr Top Microbiol Immunol.

[3] Nishikura K (2010). Functions and regulation of RNA editing by ADAR deaminases. Annu Rev Biochem.

[4] John BP, Charles ES (1995). Expression and regulation by interferon of a double-stranded-RNA-specific adenosine deaminase from human cells: evidence for two forms of the deaminase. Mol Cell Biol.

[5] Chen L, Li Y, Lin CH, Chan TH, Chow RK, Song Y, Liu M, Yuan YF, Fu L, Kong KL (2013). Recoding RNA editing of AZIN1 predisposes to hepatocellular carcinoma. Nat Med.

[6] Slotkin W, Nishikura K (2013). Adenosine-to-inosine RNA editing and human disease. GEnome Med.

[7] Zipeto MA, Jiang Q, Melese E, Jamieson CH (2015). RNA rewriting, recoding, and rewiring in human disease. Trends Mol Med.

[8] Li JB, Church GM (2013). Deciphering the functions and regulation of brain-enriched A-to-I RNA editing. Nat Neurosci.

[9] Rice GI, Kasher PR, Forte GM, Mannion NM, Greenwood SM, Szynkiewicz M, Dickerson JE, Bhaskar SS, Zampini M, Briggs TA (2012). Mutations in ADAR1 cause Aicardi-Goutieres syndrome associated with a type I interferon signature. Nat Genet.

[10] Rice GI, Forte GMA, Szynkiewicz M, Chase DS, Aeby A, Abdel-Hamid MS, Ackroyd S, Allcock R, Bailey KM, Balottin U (2013). Assessment of interferon-related biomarkers in Aicardi-Goutières syndrome associated with mutations in TREX1, RNASEH2A, RNASEH2B, RNASEH2C, SAMHD1, and ADAR: a case-control study. Lancet Neurol.

[11] Liu Q, Jiang L, Liu WL, Kang XJ, Ao Y, Sun M, Luo Y, Song Y, Lo WH, Zhang X (2006). Two novel mutations and evidence for haploinsufficiency of the ADAR gene in dyschromatosis symmetrica hereditaria. Br J Dermatol.

[12] Liu Q, Wang Z, Wu Y, Cao L, Tang Q, Xing X, Ma H, Zhang S, Luo Y. Five novel mutations in the ADAR1 gene associated with dyschromatosis symmetrica hereditaria. BMC Med Genet. 2014:15(69). 10.1186/1471-2350-15-69 PMC4105233 24950769

[13] Samuel CE (2011). Adenosine deaminases acting on RNA (ADARs) are both antiviral and proviral. Virology.

[14] Tomaselli S, Galeano F, Locatelli F, Gallo A (2015). ADARs and the balance game between virus infection and innate immune cell response. Curr Issues Mol Biol.

[15] Cuevas JM, Combe M, Torres-Puente M, Garijo R, Guix S, Buesa J, Rodriguez-Diaz J, Sanjuan R (2016). Human norovirus hyper-mutation revealed by ultra-deep sequencing. Infect Genet Evol.

[16] Shabman RS, Jabado OJ, Mire CE, Stockwell TB, Edwards M, Mahajan M, Geisbert TW, Basler CF (2014). Deep sequencing identifies noncanonical editing of Ebola and Marburg virus RNAs in infected cells. MBio.

[17] Hatakeyama M (2009). Helicobacter pylori and gastric carcinogenesis. J Gastroenterol.

[18] Westermann AJ, Gorski SA, Vogel J (2012). Dual RNA-seq of pathogen and host. Nat Rev Microbiol.

[19] Wang C, Xu JR, Liu H (2016). A-to-I RNA editing independent of ADARs in filamentous fungi. RNA Biol.

[20] Wisplinghoff H, Bischoff T, Tallent SM, Seifert H, Wenzel RP, Edmond MB (2004). Nosocomial bloodstream infections in US hospitals: analysis of 24,179 cases from a prospective nationwide surveillance study. Clin Infect Dis.

[21] Kadosh D. Control of Candida albicans morphology and pathogenicity by post-transcriptional mechanisms. Cell Mol Life Sci. 2016;73(22):4265-78. 10.1007/s00018-016-2294-y PMC5582595 27312239

[22] Duhring S, Germerodt S, Skerka C, Zipfel PF, Dandekar T, Schuster S (2015). Host-pathogen interactions between the human innate immune system and Candida Albicans-understanding and modeling defense and evasion strategies. Front Microbiol.

[23] Williams DW, Jordan RP, Wei XQ, Alves CT, Wise MP, Wilson MJ, Lewis MA. Interactions of Candida Albicans with host epithelial surfaces. J Oral Microbiol. 2013;5 10.3402/jom.v5i0.22434 PMC3805843 24155995

[24] Hebecker B, Naglik JR, Hube B, Jacobsen ID (2014). Pathogenicity mechanisms and host response during oral Candida Albicans infections. Expert Rev Anti-Infect Ther.

[25] Cassone A (2015). Vulvovaginal Candida Albicans infections: pathogenesis, immunity and vaccine prospects. BJOG.

[26] Sheppard DC, Filler SG (2015). Host cell invasion by medically important fungi. Cold Spring Harb Perspect Med.

[27] Hofs S, Mogavero S, Hube B (2016). Interaction of Candida Albicans with host cells: virulence factors, host defense, escape strategies, and the microbiota. J Microbiol.

[28] Liu Y, Shetty AC, Schwartz JA, Bradford LL, Xu W, Phan QT, Kumari P, Mahurkar A, Mitchell AP, Ravel J (2015). New signaling pathways govern the host response to C. Albicans infection in various niches. Genome Res.

[29] Ramaswami G, Lin W, Piskol R, Tan MH, Davis C, Li JB (2012). Accurate identification of human Alu and non-Alu RNA editing sites. Nat Methods.

[30] Ramaswami G, Zhang R, Piskol R, Keegan LP, Deng P, O'Connell MA, Li JB (2013). Identifying RNA editing sites using RNA sequencing data alone. Nat Methods.

[31] Yu Y, Zhou H, Kong Y, Pan B, Chen L, Wang H, Hao P, Li X (2016). The landscape of A-to-I RNA Editome is shaped by both positive and purifying selection. PLoS Genet.

[32] Kiran A, Baranov PV (2010). DARNED: a DAtabase of RNa EDiting in humans. Bioinformatics.

[33] Ramaswami G, Li JB (2014). RADAR: a rigorously annotated database of A-to-I RNA editing. Nucleic Acids Res.

[34] Picardi E, D'Erchia AM, Lo Giudice C, Pesole G (2017). REDIportal: a comprehensive database of A-to-I RNA editing events in humans. Nucleic Acids Res.

[35] Picardi E, Manzari C, Mastropasqua F, Aiello I, D’Erchia AM, Pesole G (2015). Profiling RNA editing in human tissues: towards the inosinome atlas. Sci Rep.

[36] Sakurai M, Yano T, Kawabata H, Ueda H, Suzuki T (2010). Inosine cyanoethylation identifies A-to-I RNA editing sites in the human transcriptome. Nat Chem Biol.

[37] Bahn JH, Lee JH, Li G, Greer C, Peng G, Xiao X (2012). Accurate identification of A-to-I RNA editing in human by transcriptome sequencing. Genome Res.

[38] Peng Z, Cheng Y, Tan BC, Kang L, Tian Z, Zhu Y, Zhang W, Liang Y, Hu X, Tan X (2012). Comprehensive analysis of RNA-Seq data reveals extensive RNA editing in a human transcriptome. Nat Biotechnol.

[39] Fumagalli D, Gacquer D, Rothe F, Lefort A, Libert F, Brown D, Kheddoumi N, Shlien A, Konopka T, Salgado R (2015). Principles governing A-to-I RNA editing in the breast cancer Transcriptome. Cell Rep.

[40] Han L, Diao L, Yu S, Xu X, Li J, Zhang R, Yang Y, Werner HM, Eterovic AK, Yuan Y (2015). The genomic landscape and clinical relevance of A-to-I RNA editing in human cancers. Cancer Cell.

[41] Nishikura K (2016). A-to-I editing of coding and non-coding RNAs by ADARs. Nat Rev Mol Cell Biol.

[42] Burns CM, Chu H, Rueter SM, Hutchinson LK, Canton H, Sanders-Bush E, Emeson RB (1997). Regulation of serotonin-2C receptor G-protein coupling by RNA editing. Nature.

[43] Garrett S, Rosenthal JJ (2012). RNA editing underlies temperature adaptation in K+ channels from polar octopuses. Science.

[44] Bruno VM, Shetty AC, Yano J, Fidel PL Jr, Noverr MC, Peters BM. Transcriptomic analysis of vulvovaginal candidiasis identifies a role for the NLRP3 inflammasome. MBio. 2015;6(2):e00182-15. 10.1128/mBio.00182-15 PMC4453569 25900651

[45] Kong Y, Zhou H, Yu Y, Chen L, Hao P, Li X (2015). The evolutionary landscape of intergenic trans-splicing events in insects. Nat Commun.

[46] Li H, Durbin R (2009). Fast and accurate short read alignment with burrows-wheeler transform. Bioinformatics.

[47] Li H, Handsaker B, Wysoker A, Fennell T, Ruan J, Homer N, Marth G, Abecasis G, Durbin R (2009). Genome project data processing S: the sequence alignment/map format and SAMtools. Bioinformatics.

[48] McKenna A, Hanna M, Banks E, Sivachenko A, Cibulskis K, Kernytsky A, Garimella K, Altshuler D, Gabriel S, Daly M (2010). The genome analysis toolkit: a MapReduce framework for analyzing next-generation DNA sequencing data. Genome Res.

[49] Donna K, Angela SH, Terrence SF, Krishna MR, Charles WS, David H, WJ K (2004). The UCSC table browser data retrieval tool.Pdf. Nucleic Acids Res.

[50] Chen JY, Peng Z, Zhang R, Yang XZ, Tan BC, Fang H, Liu CJ, Shi M, Ye ZQ, Zhang YE (2014). RNA editome in rhesus macaque shaped by purifying selection. PLoS Genet.

[51] Munz M, Ruark E, Renwick A, Ramsay E, Clarke M, Mahamdallie S, Cloke V, Seal S, Strydom A, Lunter G (2015). CSN and CAVA: variant annotation tools for rapid, robust next-generation sequencing analysis in the clinical setting. Genome Med.

[52] Wang K, Li M, Hakonarson H (2010). ANNOVAR: functional annotation of genetic variants from high-throughput sequencing data. Nucleic Acids Res.

[53] Quinlan AR, Hall IM (2010). BEDTools: a flexible suite of utilities for comparing genomic features. Bioinformatics.

[54] Gavin EC, Gary H, John-Marc C, Steven EB (2004). WebLogo: A Sequence Logo Generator. Genome Res.

[55] Kim D, Langmead B, Salzberg SL (2015). HISAT: a fast spliced aligner with low memory requirements. Nat Methods.

[56] Anders S, Pyl PT, Huber W (2015). HTSeq--a python framework to work with high-throughput sequencing data. Bioinformatics.

[57] Robinson MD, McCarthy DJ (2010). Smyth GK: edgeR: a bioconductor package for differential expression analysis of digital gene expression data. Bioinformatics.

[58] Bolger AM, Lohse M, Usadel B (2014). Trimmomatic: a flexible trimmer for Illumina sequence data. Bioinformatics.

[59] Chen H, Boutros PC (2011). VennDiagram: a package for the generation of highly customizable venn and euler diagrams in R. BMC Bioinformatics.

[60] Wickham H (2009). ggplot2: elegant graphics for data analysis.

